# Dose-finding study of oxaliplatin associated to capecitabine-based preoperative chemoradiotherapy in locally advanced rectal cancer

**DOI:** 10.18632/oncotarget.24665

**Published:** 2018-04-03

**Authors:** Gemma Bruera, Mario Di Staso, Pierluigi Bonfili, Antonio Galvano, Rosa Manetta, Gino Coletti, Roberto Vicentini, Stefano Guadagni, Corrado Ficorella, Ernesto Di Cesare, Antonio Russo, Enrico Ricevuto

**Affiliations:** ^1^ Oncology Territorial Care, S. Salvatore Hospital, Oncology Network ASL1 Abruzzo, University of L'Aquila, L'Aquila, Italy; ^2^ Department of Biotechnological and Applied Clinical Sciences, University of L’Aquila, L’Aquila, Italy; ^3^ Radiotherapy, S. Salvatore Hospital, Oncology Network ASL1 Abruzzo, University of L’Aquila, L’Aquila, Italy; ^4^ Medical Oncology, Department of Surgical, Oncological and Stomatological Sciences, University of Palermo, Palermo, Italy; ^5^ Radiology, S. Salvatore Hospital, L’Aquila, Oncology Network ASL1 Abruzzo, Italy; ^6^ Pathology, S. Salvatore Hospital, L’Aquila, Oncology Network ASL1 Abruzzo, Italy; ^7^ Hepatobiliar-pancreatic Surgery, S. Salvatore Hospital, L’Aquila, Oncology Network ASL1 Abruzzo, Italy; ^8^ Universitary General Surgery, S. Salvatore Hospital, Oncology Network ASL1 Abruzzo, University of L’Aquila, L’Aquila, Italy; ^9^ Medical Oncology, S. Salvatore Hospital, Oncology Network ASL1 Abruzzo, University of L’Aquila, L’Aquila, Italy

**Keywords:** capecitabine, chemoradiotherapy, dose-finding, locally advanced rectal cancer, oxaliplatin

## Abstract

**Introduction:**

Proper administration timing, dose-intensity, efficacy/toxicity ratio of oxaliplatin added to fluoropyrimidin should be improved to safely perform two-drugs intensive preoperative chemoradiotherapy in locally advanced rectal cancer (LARC). This dose-finding study investigated recommended oxaliplatin dose, safety of oxaliplatin/capecitabine regimen and preliminary activity.

**Methods:**

Schedule: oxaliplatin dose-levels, 35-40 mg/m^2^/week; capecitabine 825 mg/m^2^/ twice daily, radiotherapy on rectum/nodes, 50/45 Gy, 45 and 9 boost/45 Gy, in first 5 and subsequent patients, 5 days/week, respectively; for 5 weeks. Pathologic complete response (pCR) 10% was projected in order to positively affect clinical outcome.

**Results:**

Seventeen fit <75 years patients enrolled: median age 60; young-elderly 4 (23%); T3/T4, 15/2, N0/N1/N2, 7/9/1. At first dose-level, no dose-limiting toxicity (DLT). At second, 2 DLT, G3 mucositis, G3 thrombocytopenia, in 2/6 patients (33%). Oxaliplatin recommended dose, 40 mg/m^2^/week. Cumulative G3-4 toxicities: mucositis 6%, thrombocytopenia 6%. Limiting toxicity syndromes 18%, 25% in young-elderly, all single site. Objective response rate intent-to-treat 94%. Sphinter preservation 87%, pCR 6%. After 17 months follow-up, progression-free survival and overall survival were not reached.

**Conclusions:**

Oxaliplatin can be safely added to preoperative capecitabine-based chemoradiotherapy at the recommended dose 40 mg/m^2^/week, in LARC, with promising pCR and high activity.

## INTRODUCTION

Clinical management of locally advanced rectal cancer (LARC) faces with different options of treatment strategies according to patients’ fitness (age, performance status (PS), comorbidities), extension of primary tumor, and lymph nodes involvement. Over the last 15 years, multimodality treatments, consisting of surgery, radiotherapy and chemoradiotherapy, were evaluated, and different active drugs were used in mono and, recently, doublet chemotherapy combinations. The improving activity and efficacy of chemoradiotherapy supported preoperative sequential integration of combined modality treatment with surgical resection of LARC.

Platinum derivatives represent a class of chemotherapeutics widely used in oncology. Over the years researchers have studied the formulation of new platinum-based chemotherapeutic agents with the aim of reduce the incidence of drug related adverse events, based on the technological and biological improvement. These new compounds would be able to target specific cellular structures, carrying the pharmacological action only at the site of interest and reducing the involvement of healthy structures. Among the main examples of this strategy we find platinum A able to specifically bind the oxygenase-2 cycle (COX-2) while platinum B is able to interact with mitochondrial DNA by interfering with tumor resistance mechanisms [1].

Neo-adjuvant chemoradiotherapy, consisting of mono or doublet combinations of chemotherapeutic drugs, would not only shrink rectal tumor, but also reduce recurrence/relapse rate. 5-fluorouracil-based preoperative chemoradiotherapy association achieved pathologic complete response (pCR) 11.4%, 5-years local recurrence rate (LRR) 5-8.7%, 5-years overall survival (OS) 67.9-76%, 10-years LRR 7.1%, and 10-years OS 59.6% [2–7].

Preoperative chemoradiotherapy, consisting of 5040 cGy delivered in fractions of 180 cGy per day, five days per week, and 5-fluorouracil 1000 mg/m^2^/die during the first and fifth weeks of radiotherapy, compared with postoperative chemoradiotherapy, significantly improved local control, but did not improve OS [2, 3]. More, preoperative radiation therapy allowed 77% sphincter preservation [8]. Preoperative chemoradiotherapy, consisting of 45 Gy in 25 fractions during 5 weeks and 5-fluorouracil 350 mg/m^2^/die plus leucovorin for 5 days, at the first and fifth week, significantly improved pCR (11.4% vs 3.6%; P <.05), and local control (8.1% vs 16.5%; P <.05), despite no impact on sphincter preservation and OS [4]. In the EORTC Trial 22921, chemotherapy addition to preoperative radiotherapy induced downsizing, downstaging, and significant changes in histologic characteristics [9]. A significant benefit for local control (5-year LRR 8.7%, 9.6%, and 7.6% preoperatively, postoperatively, or both, respectively, compared to 17.1% without chemotherapy), with no significantly different OS was reported [10]. Compared to preoperative radiotherapy alone, preoperative chemoradiotherapy significantly improved pCR, 5-years LRR, but not disease-free survival (DFS) or OS [5–7], although it did not translate into a higher sphincter preservation rate [5, 7].

Short-term regimen of high-dose preoperative radiotherapy (25 Gy in five fractions of 5 Gy), compared to surgery alone, significantly reduced LRR, and increased 5-year OS (58%) [11]. Early radiation toxicity was higher with chemoradiotherapy compared to short-term radiotherapy (18.2%), with no different LRR and clinical outcome [12, 13]. Long-course preoperative chemoradiation resulted in significantly greater tumour downsizing and downstaging compared with short-term radiation, with no difference in R0 resection rates [14].

In a randomised, non-inferiority, phase 3 trial, comparing capecitabine- with 5-fluorouracil-based chemoradiotherapy, clinical outcome with capecitabine was non-inferior [15], but fewer patients developed distant metastases. No significant differences in pCR, sphincter-saving surgery, or surgical downstaging were reported [16].

The major challenge of the addition of more drugs in a chemotherapy combination is the proper design of schedule and dose, providing the balance between dose intensity (DI) of each drug and safety. Oxaliplatin and 5-fluorouracil- or capecitabine-based chemoradiotherapy was performed at different doses and schedules with significantly increased toxicity in randomized studies, limiting not only the favourable impact of the association, but, also, the expected effectiveness of radiotherapy [17–20]. Oxaliplatin addition achieved pCR 16-19.2%, 3-years LRR 4.4%, and 3-years OS 88.3%. Significantly increased pCR was demonstrated only in the CAO/ARO/AIO-04 trial [19]. More active doublet chemotherapy seems to require continuous 5-fluorouracil infusion 250 mg/m^2^/die days 1-14 and 22-35, and oxaliplatin at projected DI (pDI) 40 mg/m^2^/w, respectively. Other schedules failed to significantly demonstrate improved activity and clinical efficacy.

A standard preoperative chemoradiotherapy regimen (50.4 Gy in 28 daily fractions with 5-fluorouracil infusion 225 mg/m^2^/die) was compared with the addiction of oxaliplatin 60 mg/m^2^/week for 6 weeks, in a phase III trial [17]: pCR rate was 16% in both arms (P =.904). In the ACCORD 12 trial, preoperative chemoradiotherapy, 45 Gy in 25 fractions for 5 weeks with capecitabine 800 mg/m^2^/twice daily (CAP45) was compared with 50 Gy radiotherapy in 25 fractions in association to capecitabine and oxaliplatin 50 mg/m^2^/week for 5 weeks (CAPOX50) [18]: pCR were trendly increased up to 19.2% (P =.09), with no significantly different 3-years LRR and clinical outcome. In the CAO/ARO/AIO-04 phase III trial [19], preoperative radiotherapy 50.4 Gy plus infusional 5-fluorouracil (250 mg/m^2^ days 1-14 and 22-35) and oxaliplatin (50 mg/m^2^ days 1,8,22,29) significantly increased pCR rate (17%). In the NSABP trial, preoperative radiotherapy (4500 cGy in 25 fractions over 5 weeks plus boost of 540cGy-1080cGy in 3-6 fractions) associated to continuous infusion 5-fluorouracil (225 mg/m^2^ 5 days/week), or capecitabine (825 mg/m^2^/ twice daily 5 days/week), with or without oxaliplatin (50 mg/m^2^/week for 5 weeks) [20], did not show significant differences in LRR, DFS, or OS between 5-fluorouracil-capecitabine or oxaliplatin-none.

The present dose-finding study, proposing preoperative chemoradiotherapy combining oxaliplatin and capecitabine for LARC patients, assess oxaliplatin dose to be recommended for prospective clinical trials, safety and preliminary activity.

## RESULTS

### Patient demographics

Seventeen consecutive patients were enrolled (Table 1): Male/Female ratio, 12/5; median age, 60 years; 4 (23%) young-elderly (≥ 65 <75 y); Cumulative Illness Rating Scale (CIRS) [21] primary 5 (29%), intermediate 12 (71%); all WHO PS 0; clinical tumor stage T3, 15 (88%), T4, 2 (12%), lymph nodes stage N0, 7 (41%), N1, 9 (53%), N2, 1 (6%).

**Table 1:** Patients’ features

	Total N. (%)
No. of patients	17
Sex	
Male/Female	12/5
Age, years	
Median	60
Range	39-69
≥ 65 years	4 (23)
CIRS stage	
Primary	5 (29)
Intermediate	12 (71)
Secondary	-
WHO Performance Status	
0	17 (100)
1-2	-
Tumor stage	
T1	-
T2	-
T3	15 (88)
T4	2 (12)
Lymph nodes stage	
N0	7 (41)
N1	9 (53)
N2	1 (6)

Abbreviation: CIRS, Cumulative Illness Rating Scale; WHO, World Health Organization.

### Dose finding

At the first dose-level, 3 patients were enrolled and 15 weeks of treatment were administered; no DLT was observed out of 3 patients. At the second dose level, 14 patients were treated (2 cohorts of 3 patients, and other 8 patients). A DLT, G3 mucositis, was observed in 1 out of 3 patients (33%) of the first cohort; in the second cohort of 3 patients, a DLT, G3 thrombocytopenia, was observed. Thus, 2 DLTs were observed out of 6 patients (33%) and out of 30 weeks of treatment (10%). Thus, oxaliplatin maximum tolerated dose was reached at the second dose level (Table 2), and oxaliplatin 40 mg/m^2^/week was the recommended dose. Eight more patients were treated at the recommended dose, with 1 limiting toxicity (G2 mucositis > than 2 weeks). Thus, at the recommended dose DLT were 3 out of 14 patients (21%).

**Table 2:** Oxaliplatin dose-finding

Dose levels	Capecitabine (mg/m^2^/ twice daily 5d on 2d off)-Oxaliplatin (mg/m^2^/week)	No. Patients^a^	No. weeks	No. patients with DLT/total patients (%)	No. cycles with DLT/total weeks (%)	DLTs
I	825-35	3	15	-/3(-)	-/15(-)	-
II	825-40	6	30	2/6 (33%)	3/30 (10%)	G3 Mucositis G3 Thrombocytopenia

^a^Inter-patient dose escalation;

Abbreviation: DLT, Dose-Limiting Toxicity.

Among 12 patients treated with 45 Gy in 25 daily fractions of 1.8 Gy on rectal tumour and locoregional lymph nodes and boost of 9 Gy in 25 daily fractions of 0.36 Gy on rectal tumor, 1 hematological toxicity was reported, with 1 local toxicity (G2 mucositis > than 2 weeks). Thus, radiotherapy 54 Gy on rectal tumor, and oxaliplatin 40 mg/m^2^/week, can be concomitantly administered as preoperative chemoradiotherapy in LARC.

### Dose-intensity

Median number of weeks of treatment was 5 (range 2-5). Median rDI per patient were: capecitabine 6187.5 (4125-8250) mg/m^2^/week, 75% of pDI; oxaliplatin 27.5 (20-40) mg/m^2^/week, 68.75% of pDI. In 4 young-elderly patients, median rDIs per cycle were 100% of pDI.

### Toxicity

Table 3 describes cumulative toxicities in 17 enrolled patients and in 82 administered weeks. One out of 17 patients (6%) discontinued capecitabine/oxaliplatin treatment due to limiting toxicity (LT) (grade 3 thrombocytopenia). Cumulative G3-4 toxicities, by patients, were: mucositis 1 (6%), thrombocytopenia 1 (6%), in a young-elderly and a non-elderly patient, respectively. Cumulative G2 toxicities, by patients, were: diarrhoea 4 (23%), constipation 1 (6%), mucositis 2 (12%), asthenia 1 (6%). No case of thrombosis, hemorrhage/bleeding, cardiac or cerebrovascular ischemia, G4 neutropenia, febrile neutropenia, G4 thrombocytopenia, or toxic deaths were observed.

**Table 3:** Cumulative toxicity

	Patients				Weeks			
*Number*	17				82			
*NCI-CTC Grade*	1	2	3	4	1	2	3	4
Nausea (%)	3 (18)	-	-	-	7 (8)	-	-	-
Vomiting (%)	1 (6)	-	-	-	1 (1)	-	-	-
Diarrhea (%)	6 (35)	4 (23)	-	-	17 (21)	4 (5)	-	-
Hypoalbuminemia (%)	-	-	-	-	-	-	-	-
Constipation (%)	-	1 (6)	-	-	-	3 (4)	-	-
Mucositis (%)	10 (59)	2 (12)	1 (6)	-	27 (33)	4 (5)	2 (2)	-
Erythema (%)	-	-	-	-	-	-	-	-
Asthenia (%)	8 (47)	1 (6)	-	-	19 (23)	2 (2)	-	-
Neurotoxicity (%)	5 (29)	-	-	-	11 (13)	-	-	-
Hypertension (%)	-	-	-	-	-	-	-	-
Hypotension (%)	-	-	-	-	-	-	-	-
Hematuria (%)	-	-	-	-	-	-	-	-
Gengival recession/gengivitis (%)	-	-	-	-	-	-	-	-
Rhinitis (%)	-	-	-	-	-	-	-	-
Epistaxis (%)	-	-	-	-	-	-	-	-
Hand-foot skin reaction (%)	-	-	-	-	-	-	-	-
Hypokalemia (%)	-	-	-	-	-	-	-	-
Hypertransaminasemy (%)	1 (6)	-	-	-	1 (1)	-	-	-
Hyperpigmentation (%)	-	-	-	-	-	-	-	-
Fever without infection (%)	-	-	-	-	-	-	-	-
Alopecia (%)	-	-	-	-	-	-	-	-
Anemia (%)	-	-	-	-	-	-	-	-
Leucopenia (%)	-	-	-	-	-	-	-	-
Neutropenia (%)	-	-	-	-	-	-	-	-
Thrombocytopenia (%)	-	-	1 (6)	-	-	-	1 (1)	-

Abbreviation: NCI-CTC, National Cancer Institute Common Toxicity Criteria.

Overall, limiting toxicity syndromes (LTS) [22, 23] were observed in 3 patients (18%); 1 out of 4 young-elderly patients (25%), all single-site limiting toxicity syndromes (LTS-ss), characterized by G3 mucositis, G3 thrombocytopenia, and G2 mucositis for > than 2 weeks.

### Activity and efficacy

Overall, 17 patients were enrolled (Table 4). In the intent-to-treat analysis 16 patients were evaluable: Objective response rate (ORR) was 94% (α 0.05, CI ± 12). We observed 15 objective partial responses; 1 liver progressive disease (6%) with local partial response. In the as-treated analysis, 16 patients were evaluable: 1 patient did not received the planned 5 weeks of treatment, due to haematological LT requiring chemotherapy discontinuation. ORR was 94% (α 0.05, CI ± 12). We observed 15 objective partial responses; 1 progressive disease (6%). After a median follow-up of 17 months, median PFS was not reached (2-67+): 5 events occurred (local recurrence 1 patient, 6%). Median OS was not reached (2+-67+): 1 event occurred. No significant association were reported with *RAS* or *BRAF* mutational status.

**Table 4:** Activity and efficacy data

	Intent-to-treat analysis		As-treated analysis	
	No	%	No	%
**Enrolled patients**	17	100	17	100
**Evaluable patients**	16	94	16	94
**Objective response**	15	94 (CI ± 12)	15	94 (CI ± 12)
Partial response	15	94	15	94
Complete response	-	-	-	-
**Stable disease**	-	-	-	-
**Progressive disease**	1	6	1	6
**Median progression-free survival, months**	not reached			
Range	2-67+			
Progression events	5	29		
**Median overall survival, months**	not reached			
Range	2+-67+			
Deaths	1	6		
**Sphinter preserving surgery (No./patients who underwent surgery)**	13/15	87		
**Pathologic complete response (No./patients who underwent surgery)**	1/15	7		

Sphincter preserving surgery was performed in 13 of 15 patients who underwent surgery (87%); 1 pCR was achieved (7%), in a patient progression-free at 20 months. All R0 resections were performed. No post-operative toxicity or mortality were reported.

Approximately half patients (7) received adjuvant treatment with capecitabine / oxaliplatin (5) or capecitabine alone (2) regimens. Three patients developed disease progression and were treated with the first-line FIr-B/FOx association [22], other two patients underwent cetuximab-containing associations. Second-line treatment options were capecitabine/bevacizumab (1) and treatment with cetuximab (1).

## DISCUSSION

The present dose-finding study proposing oxaliplatin and capecitabine doublet chemotherapy association combined with preoperative radiotherapy, recommended oxaliplatin dose 40 mg/m^2^/week for safely administration in clinical trials to properly evaluate its contribution to clinical outcome in LARC patients. Oxaliplatin and capecitabine association was feasible at median oxaliplatin rDI 68.75% (27.5 mg/m^2^/week) and capecitabine rDI 75% (6187.5 mg/m^2^/week). Cumulative G3-4 toxicities were represented by mucositis (6%), and thrombocytopenia (6%). Three individual LTS, all LTS-ss, were reported in 18% patients. G2 toxicities were represented by diarrhea 23%, mucositis 12%, asthenia 6%. In the CAO/ARO/AIO-04 phase III trial [19], grade 3-4 toxicities occurred in 23% patients who received oxaliplatin (50 mg/m^2^ days 1, 8, 22, and 29) added to infusional 5-fluorouracil (1000 mg/m^2^ days 1-5 and 29-33) and radiotherapy (50.4 Gy), compared to 20% with 5-fluorouracil. More common grade 3-4 toxicities in 5-fluorouracil/oxaliplatin arm were: diarrhoea (12% vs 8%), nausea/vomiting (4% vs 1%). In the experimental and control arms, full dose of chemotherapy and radiotherapy was administered in 85% and 79%, 94% and 96% of patients, respectively.

In a randomised, phase 3 trial, comparing capecitabine-based with 5-fluorouracil-based chemoradiotherapy [15], diarrhoea was the most common adverse event in both groups (any grade 53% vs 44%, grade 3-4 9% vs 2%, respectively); hand-foot skin reactions (31% vs 2% any grade, 2% vs none grade 3-4), fatigue (28% vs 15% any grade, none vs 1% grade 3-4), and proctitis (16% vs 5% any grade, <1% vs <1% grade 3-4), were more frequent with capecitabine; while leucopenia with 5-fluorouracil (35% vs 25% any grade, 8% vs 2% grade 3-4).

In most reported prospective randomized trials G3-4 toxicity was significantly increased in treatment arm including oxaliplatin: 24% in the STAR01; 25% in the ACCORD 12; 15% in the NSABP R-04 [18]. They were mainly represented by: diarrhea 15.3% and 12.3%, and similarly with capecitabine [15]; radiation dermatitis 4.5%, asthenia 3.1%, neurotoxicity 1.4%, nausea/vomiting 4% [17, 19]. In the NSABP trial, oxaliplatin addition (50 mg/m^2^/week for 5 weeks) to continuous infusion 5-fluorouracil (225 mg/m^2^ 5 days/week) or capecitabine (825 mg/m^2^/ twice daily 5 days/week) and radiotherapy (45 Gy) was associated with significantly more grade 3-4 diarrhea (P < 0.001) [20]. In the STAR-01 trial [17], among patients treated with oxaliplatin 60 mg/m^2^/weekly, added to standard preoperative 5-fluorouracil 225 mg/m^2^/die chemoradiotherapy (50.4 cGy), 66% received the six planned weekly oxaliplatin administrations, 75% received at least 80% of the planned cumulative oxaliplatin dose, and significantly less patients received the planned fluoropyrimidine dose (80% vs 90%; P <.001), and the full planned radiotherapy dose (84% vs 92%; P <.001). Treatment discontinuation rate due to toxicity was 17%. Limiting diarrhea (15.3%), radiation dermatitis (4.5%), asthenia (3.1%) were significantly more common in oxaliplatin arm. Grade 3 neurotoxicity was 1.4%.

One of the major reason that can justify the failure of the association of oxaliplatin to 5-fluorouracil or capecitabine to significantly increase activity and efficacy of LARC patients can be ascribed to toxicity limiting the realization of the chemoradiotherapy strategy. Our preliminary data concerning activity and efficacy show that the present schedule of safely administration of capecitabine/oxaliplatin associated to radiotherapy may achieve 87% radical resection of LARC with sphinter preservation, 7% ypCR, median PFS not reached. 5-fluorouracil-based preoperative chemoradiotherapy achieved pCR 11.4%, 5-years LRR 5-8.7%, 5-years OS 67.9-76%, 10-years LRR 7.1%, and 10-years OS 59.6% [2–7]. In ‘fit’ patients, chemoradiotherapy integrated with secondary resection of rectal tumor, significantly reduced LRR, and increased survival over surgery alone. Addition of oxaliplatin to 5-fluorouracil- or capecitabine-based preoperative chemoradiotherapy represents a step forward to intensify medical preoperative treatment of LARC patients. Doublets [17–20] achieved pCR 16-19.2%, significantly increased in the CAO/ARO/AIO-04 trial [19], with 3-years LRR 4.4%, and 3-years OS 88.3%, although a higher sphincter preservation rate, local control and efficacy were not achieved. In the subgroup of patients achieving ypT0, oxaliplatin addition significantly increased 3-year DFS, compared to the control arm [18].

Definition of the proper schedule to safely integrate oxaliplatin and capecitabine doublet chemotherapy with radiotherapy represents a mainstay to further investigate in randomized clinical studies whether more intensive medical treatments could increase pCR rate, local recurrence control, and clinical outcome in LARC patients.

The present dose-finding study proposed a feasible and safe schedule of doublet capecitabine and oxaliplatin association, at oxaliplatin recommended dose 40 mg/m^2^/week, that should be evaluated in prospective trials in LARC patients.

## MATERIALS AND METHODS

### Patient eligibility

Patients were eligible if they had histologically confirmed diagnosis of measurable LARC, clinically staged as cT3/T4 any cN or any cT cN1/N2; age 18-75 years; World Health Organization (WHO) PS ≤ 2; adequate hematological, renal and hepatic functions; life expectancy more than 3 months.

CIRS was used to evaluate the comorbidity status, and only patients with primary and intermediate CIRS stage were enrolled [21]. Primary CIRS stage consisted of: independent Instrumental Activity of Daily Living (IADL), and absent or mild grade comorbidities; intermediate CIRS stage consisted of dependent or independent IADL, and < 3 mild or moderate grade comorbidities. Patients with secondary CIRS stage, consisting of ≥ 3 comorbidities or a severe comorbidity, with or without dependent IADL, were not enrolled. Criteria to define patients unfit for the proposed treatment strategy were: uncontrolled severe diseases; cardiovascular disease (uncontrolled hypertension, uncontrolled arrhythmia, ischemic cardiac diseases in the last year); thromboembolic disease, coagulopathy, preexisting bleeding diatheses.

Sequential chemoradiotherapy with capecitabine and oxaliplatin, and rectal surgery was proposed to consecutive eligible LARC patients as a treatment strategy in clinical practice, chosen among those in indication and approved by Agenzia Italiana del Farmaco (AIFA) for administration *in label* in Italian public hospitals, and published in Gazzetta Ufficiale Repubblica Italiana (“Elenco dei Medicinali erogabili a totale carico del Servizio Sanitario Nazionale”, Gazzetta Ufficiale Repubblica Italiana N.1, 2 Gennaio 2009). Treatments and schedules have been used in common clinical practice, no approval by ethics committee and institutional review board was required. All patients provided written, informed consent to the proposed *in label* treatment strategy. Treatment was conducted in accordance with the Declaration of Helsinki.

### Methods

#### Schedule

It was a dose-finding study evaluating safety, activity and efficacy of doublet chemotherapy association, consisting of capecitabine and oxaliplatin, combined with radiotherapy, as preoperative treatment in LARC patients. Doublet chemotherapy association was administered according to the following schedule: capecitabine (Xeloda, Roche, Welwyn Garden City, United Kingdom), per os, at the dose of 825 mg/m^2^/ twice daily 5 days/week; oxaliplatin (Eloxatin; Sanofi-Aventis, Milan, Italy), over 2-hours as a 250 ml intravenous infusion of a solution containing 5% glucose, at the dose of 35-40 mg/m^2^/week. The concentration of the capecitabine used is already enstabilished in neoadjuvant setting trials, 825 mg / m2 twice daily for 5 days as radiosensitizing agent.

Radiation therapy in 25 daily fractions, 5 days a week: 50 Gy of 2 Gy on rectal tumor and 45 Gy of 1.8 Gy on locoregional lymph nodes, in the former 5 patients; 45 Gy of 1.8 Gy on rectal tumour and locoregional lymph nodes and boost of 9 Gy in 25 daily fractions of 0.36 Gy on rectal tumor, in the latter 12 patients.

#### Study design

Physical examination and routine laboratory tests were performed at baseline and every week on-treatment, including complete blood cell count, electrolytes, liver and renal function, urine examination and coagulation function; tumor markers every 4 weeks; electrocardiogram every four weeks and echocardiogram at baseline, and at the end of treatment.

Primary end-point was to define the recommended oxaliplatin dose. Secondary end-points were evaluation of toxicity, pCR rate, ORR, progression-free survival (PFS), OS. Toxicity was registered every week according to National Cancer Institute Common Toxicity Criteria (version 3.0). Dose-limiting toxicity (DLT) was defined as grade 3-4 non-haematological toxicity (mainly represented by diarrhea, mucositis, neurotoxicity, hand-foot syndrome, asthenia), grade 4 hematologic toxicity (leuconeutropenia), febrile neutropenia, grade 3-4 thrombocytopenia, or any toxicity determining > 2 weeks treatment delay. To discriminate individual safety, LTS, consisting of at least a limiting toxicity (LT) associated or not to other limiting or G2 toxicities, were evaluated, as previously reported [22, 23]. LTS were classified as LTS single site (LTS-ss), characterized only by the LT, and LTS multiple sites (LTS-ms), ≥ 2 LTs or a LT associated to other, at least G2, non-limiting toxicities.

ORR was evaluated according to RECIST criteria [24]; pCR was defined as absence of residual cancer cells in surgically resected specimens; PFS and OS, using Kaplan-Meier method [25]. PFS was defined as length of time between the beginning of treatment and disease progression or death (resulting from any cause) or to last contact; OS as length of time between the beginning of treatment and death or to last contact.

Patients were evaluated at baseline and after treatment by a multidisciplinary team, consisting of medical oncologist, radiotherapist, surgeon, and radiologist, to dynamically evaluate multimodality treatment strategy. Resection of primary rectal tumor was defined R0, if radical surgery, R1, if microscopic residual cancer cells were present at resection margins. Surgery was recommended > 6-8 weeks after chemo radiotherapy discontinuation.

Clinical evaluation of response was planned by rectoscopy, CT-scan, and transrectal magnetic resonance; PET was added based on investigators’ assessment. Follow-up was scheduled every three months up to disease progression or death.

### Statistical design

This dose-finding study was developed to verify recommended oxaliplatin dose, by 2 escalating steps at 35 and 40 mg/m^2^, according to an inter-patient approach [26]. It is preliminary to a phase II study, evaluating activity and efficacy of doublet chemotherapy associated to preoperative radiotherapy, assuming as minimal interesting activity a rate of pCR 10% [27]. For the study design, we used a two stage Simon procedure.

## Abbreviations

CIRS: Cumulative Index Rating Scale
DFS: Disease-free survival
DLT: Dose-limiting toxicity
IADL: Instrumental Activity of Daily Living
LARC: Locally advanced rectal cancer
LRR: Local recurrence rate
LTS: Limiting toxicity syndromes
LTS-ss: Limiting toxicity syndromes single-site
ORR: Objective response rate
OS: Overall survival
pCR: Pathologic complete response
PFS: Progression-free survival
PS: Performance status
rDI: received dose-intensity
WHO: World Health Organization
